# Nuclear VAV1 increases GLI1-dependent transcription in pancreatic cancer cells

**DOI:** 10.1016/j.jbc.2025.110988

**Published:** 2025-11-26

**Authors:** Brooke R. Tader, Luciana L. Almada, Murat Toruner, Kayla C. LaRue-Nolan, David L. Marks, Ashley N. Sigafoos, Laura Hilario-Garcia, Vladimir G. Gainullin, Daniel D. Billadeau, Martin E. Fernandez-Zapico

**Affiliations:** 1 Division of Oncology Research, Department of Oncology, Mayo Clinic, Rochester, Minnesota, USA; 2 Mayo Clinic Graduate School of Biomedical Sciences, Mayo Clinic, Rochester, Minnesota, USA

**Keywords:** VAV1, GLI1, pancreatic cancer, nuclear translocation, gene transcription

## Abstract

The oncogenic role of VAV1, a GTPase guanine nucleotide exchange factor (GEF) with cytoplasmic and nuclear localizations, has been previously reported in multiple malignancies. Most of the mechanisms underlying this pro-tumoral activity have been linked to the cytoplasmic expression of this GEF. To date, the contribution of nuclear VAV1 to cancer development remains poorly understood. Here, using models of pancreatic ductal adenocarcinomas (PDAC), the most common subtype of pancreatic cancer, we provide evidence of a novel mechanism driving oncogenic gene expression in PDAC cells that is regulated by nuclear VAV1. We show that VAV1 wild-type, unlike its mutant lacking the nuclear localization signal (NLS), localizes to the nucleus of PDAC cells where it increases GLI transcriptional activity without affecting the expression of GLI factors (GLI1, GLI2 and GLI3). Interestingly, this VAV1 NLS-deficient mutant loses interaction with Importin b1 but maintains ability to activate RAC1. Further analysis showed that VAV1 and GLI1 endogenously interact in PDAC cells, and knockdown of VAV1 reduces the expression of a set of GLI target genes including BCL2. We found VAV1 bound to the GLI binding motif present within the *BCL2* promoter region and demonstrate the requirement of VAV1 to maintain *BCL2* expression and promoter activity. Finally, we showed that VAV1 is necessary for the binding of GLI1 and its coactivator the histone acetyltransferase PCAF to this regulatory element. Taken together, our data supports a role for VAV1 in GLI1 transcriptional regulation, elucidating a new mechanism of function for nuclear VAV1 in PDAC cells.

VAV1 is a Rho family guanine nucleotide exchange factor (GEF), which activates downstream GTPases by catalyzing the exchange of GDP for GTP. VAV1, through its GEF activity, can activate Rho family GTPases, such as Rac1, leading to remodeling of the actin cytoskeleton as well as downstream c-Jun N-terminal kinase (JNK) activation (1, 2). VAV1 also has GEF-independent functions, mostly described in hematopoietic cells, including activating transcription factors, such as NFAT and AP-1, where VAV1 can serve to integrate signaling to drive the activation of these transcription factors, including regulating calcium flux *via* phospholipase c-γ (3, 4, 5, 6). Other GEF-independent functions include regulating thymocyte selection *via* apoptosis (7) and the recruitment of SOS1 and SOS2 to the plasma membrane–associated adapter protein LAT, which enhances RAS and PI3K signaling (6). Ectopic expression of VAV1 occurs in a variety of cancer types, including pancreatic ductal adenocarcinoma (PDAC) (8), breast cancer (9), gastric cancer (10), esophageal squamous cell carcinoma (11), and others, and is often associated with poorer survival rates. In PDAC, ectopic VAV1 expression occurs *via* demethylation in VAV1’s promoter region, leading to an increase in cell proliferation (8), as well as an increase in both invadopodia and lamellipodia formation, ultimately promoting invasion and migration (12, 13). Further understanding of the mechanism underlying the oncogenic function of VAV1 is of great biological importance as it can help improve future therapeutic efforts for cancer patients.

In this study, we demonstrate a novel functional interaction between VAV1 and GLI1, a member of the Kruppel family of zinc finger–containing transcription factors. GLI1 is best known for its role as a downstream effector of Hedgehog (Hh) signaling, promoting transcription of target genes driving proliferation and survival (14). GLI transcription factors can also be activated in the absence of Hh ligands. This noncanonical activation of GLI1 occurs in several cancers, including PDAC (15, 16). Separately, both GLI1 and VAV1 have been shown to be required for PDAC tumor cell proliferation, and the presence of either protein in PDAC has been associated with poor prognosis (8, 15). Here, we show that VAV1 can localize to the nucleus of PDAC cells, and we determined that nuclear VAV1 is required to exert its positive regulatory effect on GLI1 in PDAC cells. We also demonstrate physical interaction of VAV1 and GLI1 in the nucleus of PDAC cells. Chromatin immunoprecipitation (ChIP) studies show the binding of both proteins to the promoter region *BCL2*, a known target of GLI1, and demonstrate the requirement of VAV1 for the binding of GLI1 to this regulatory region and acetyltransferase PCAF. This study expands our understanding of the role of VAV1 in neoplastic transformation and allows us to gain new insight into the network of signaling pathways involved in the pathogenesis of PDAC.

## Results

### VAV1 localizes to the nucleus of PDAC cells

**Figure 1:**
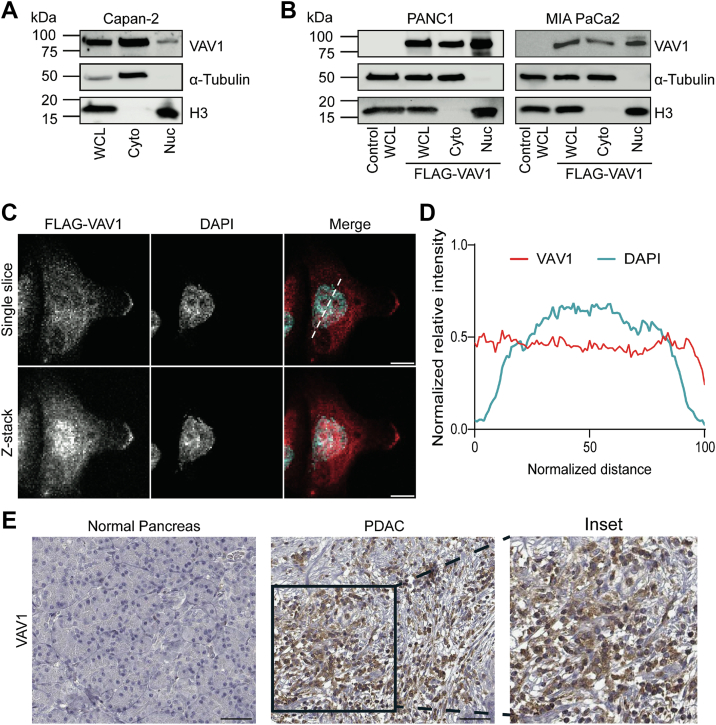
**VAV1 subcellular localization in PDAC cells**. *A*, representative Western blot images of nuclear fractions from Capan-2 cells indicating the presence of endogenous VAV1 in both cytoplasmic and nuclear fractions (n = 3). *B*, representative Western blot images of a nuclear fractionation were performed in FLAG-VAV1–transfected PANC1 (*left*) and MIA PaCa2 (*right*) cells indicating the presence of VAV1 in cytoplasmic and nuclear fractions (n = 3). *C*, representative single-slice and z-stack confocal microscopy image of FLAG in PANC1 cells expressing a FLAG-tagged WT VAV1 construct (n = 3; the scale bar represents 10 μm). The *dashed line* in *C* indicates where the line scan analysis was performed. *D*, line scan analysis of FLAG-VAV1 and DAPI signal from (*C*) showing overlap of fluorescence between VAV1 and DAPI in PANC1 cells (n = 30). *E*, IHC for VAV1 in human tissue from a normal pancreas sample (*left*), with no VAV1 staining present, and from a PDAC sample *(middle*), with an *inset* of the PDAC sample (*right*) showing staining for VAV1 present in both nuclear and cytoplasmic compartments of PDAC cells (the scale bar represents 50 μm). Cyto, cytoplasmic; DAPI, 4′,6-diamidino-2-phenylindole; IHC, immunohistochemistry; Nuc, nuclear; PDAC, pancreatic ductal adenocarcinoma; WCL, whole-cell lysate.

Initially, nuclear fractionation was performed, followed by Western blotting for VAV1, α-tubulin (cytoplasmic marker), and total H3 (nuclear marker) to determine the localization of endogenous VAV1 in Capan-2 (Fig. 1*A*), Panc 05.04, and Panc 04.03 (Fig. S1*A*) cell lines. We were able to show that endogenous VAV1 primarily localizes to the cytoplasm but is also found in the nucleus in all three lines. Overexpression of FLAG-tagged WT VAV1 in a VAV1-negative and low VAV1-expressing cell line, PANC1 and MIA PaCa2, respectively, showed the presence of VAV1 in both cellular compartments, with a larger fraction of overexpressed VAV1 present in the nuclear compartment following nuclear fractionation (Fig. 1*B*). Immunofluorescence (IF) following FLAG-VAV1-WT expression in PANC1 cells also indicates the presence of VAV1 throughout the cytoplasm and nucleus, as quantified by line scan analysis (Fig. 1, *C* and *D*). These results were confirmed using an mCherry-tagged VAV1-WT expression construct in PANC1 cells and quantified by line scan analysis (Fig. S1, *B*–*D*). Furthermore, we determined whether the localization of VAV1 was influenced by culture serum, a known regulator of its activity (17, 18). To this end, we performed subcellular localization assays of VAV1 in PANC1 cells transfected with FLAG-VAV1-WT and subjected to serum deprivation for 24 and 48 h. Other experimental groups included cells that were not subjected to serum deprivation or that were deprived of serum for 24 h, followed by the reintroduction of serum for another 24 h before analysis. Nuclear fractionation did not reveal significant changes in the nucleus-to-cytoplasm ratio in any of the starved/reinduced groups. Notably, VAV1 expression consistently favored nuclear localization, even under conditions of serum deprivation (Fig. S1*E*). Finally, immunohistochemistry (IHC) images of human tissue samples obtained from the Human Protein Atlas (17) show no staining of VAV1 in a normal pancreas, whereas a PDAC tissue sample showed staining for VAV1, with signal occurring in both nuclear and cytoplasmic compartments of tumor cells (Fig. 1*E*). Mutational analysis of five independent PDAC cohorts found that the number of VAV1 alterations or mutations represents only a total of 1.1% across close to 1000 PDAC patients; therefore, the aberrant increased expression of VAV1 in PDAC most likely occurs in its WT form. These findings show that VAV1 can localize to the nucleus of PDAC cells both *in vitro* and *in vivo*, which could indicate a novel mechanism driving the oncogenic function of VAV1 in PDAC.

### VAV1 is a positive regulator of GLI1

**Figure 2:**
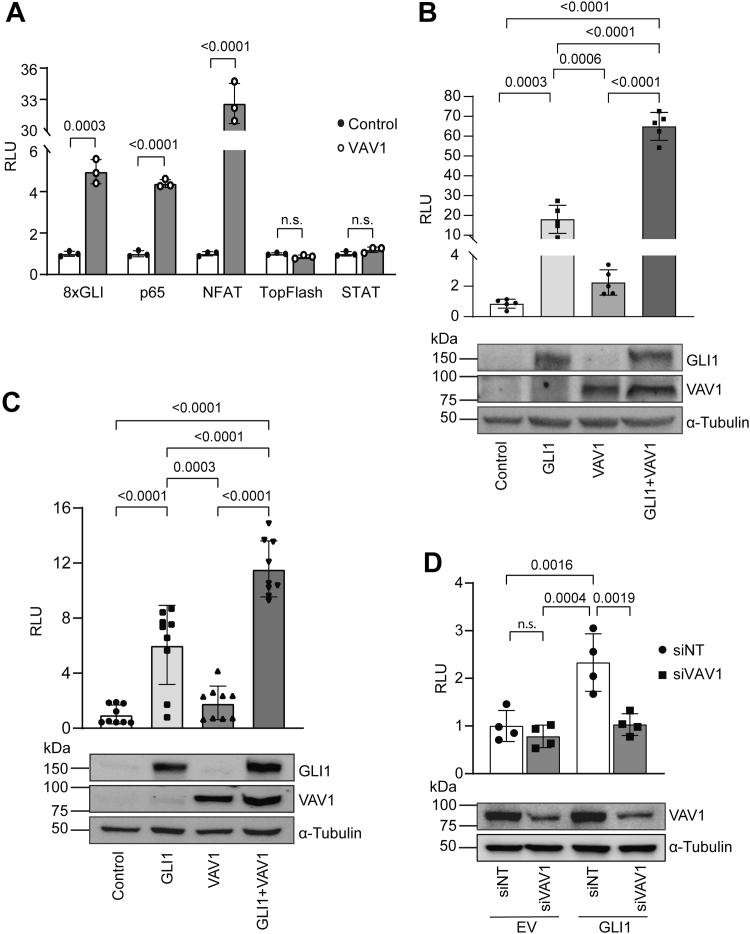
**VAV1 exerts a positive regulatory effect on GLI transcriptional activity**. *A*, luciferase assay was performed in MIA PaCa2 cells showing the effect of VAV1 overexpression on the activity of transcription factors (n = 3) with a known role in PDAC biology. Unpaired, two-tailed *t* tests were performed for each luciferase reporter comparing VAV1 overexpression to an empty vector control group. *B*, luciferase assay performed in MIA PaCa2 cells transfected with the 8xGLI reporter, along with GLI1, VAV1. To minimize saturation of the experiment, VAV1 was transfected at one-fourth the amount used in panel (*A*)] or a combination of both constructs (*top panel*; n = 5). One-way ANOVA was performed with a post hoc Tukey’s multiple comparison test. Representative Western blot images showing expression of FLAG-tagged GLI1 and VAV1 (*bottom panel*). *C*, luciferase assay was performed in PANC1 cells using an 8xGLI reporter and the constructs shown (*top panel*; n = 9). One-way ANOVA was performed with a post hoc Tukey’s multiple comparison test. Representative Western blot images showing expression of FLAG-tagged GLI1 and VAV1 (*bottom panel*). *D*, luciferase assay was performed in Panc 04.03 cells following knockdown of VAV1 *via* siRNA and the overexpression of GLI1. One-way ANOVA was performed with a post hoc Tukey’s multiple comparison test. Representative Western blot image of VAV1 with α-tubulin loading control. All data points indicate the average value for independent biological replicates. PDAC, pancreatic ductal adenocarcinoma.

VAV1 has been shown to increase transcriptional activation of NFAT and NF-κB in rat basophilic leukemia cells (4); therefore, we wanted to investigate if the activation of these factors by nuclear VAV1 also contributes to its oncogenic function in PDAC cells. Importantly, these factors are known to participate in PDAC tumorigenesis (18, 19, 20, 21, 22). For instance, p65, a member of the NF- κB family of transcription factor complexes, has been shown to be essential for the progression of preneoplastic lesions (PanIN) to PDAC, as well as promoting PDAC invasiveness (20, 21, 22). Similarly, NFAT factors, especially NFATc1, have been shown to promote proliferation and invasion of PDAC cells (18, 19). In addition to these factors, we screened reporters for other transcription factors with known oncogenic function in PDAC cells, including β-catenin (TOPFlash), GLI (8xGLI), and STAT (15, 23, 24, 25, 26). We performed luciferase reporter assays in MIA PaCa2 cells to determine if VAV1 changes the activity of these transcription factors (Fig. 2*A*). As expected, VAV1 increased the transcriptional activity of NFAT and p65 reporters but did not affect the activation of STATs or the β-catenin reporter. Interestingly, VAV1 overexpression increased the activity of the GLI reporter, which has not been previously shown.

The GLI family of transcription factors includes GLI1, GLI2, and GLI3, where GLI1 is primarily a transcriptional activator. GLI2 and GLI3 can function as transcriptional repressors or activators, depending on the cellular context (27, 28). To determine whether the effect of VAV1 overexpression is mediated by an increased expression of GLI transcription factors, we performed RT–quantitative PCR (qPCR) following overexpression of VAV1 and showed that the relative mRNA expression of *GLI1*, *GLI2*, and *GLI3* was unchanged in the presence of VAV1 compared with the empty vector (EV) control (Fig. S2*A*). Since VAV1 increased GLI transcriptional activity and GLI1 is the major transcriptional activator in this family, we next evaluated if GLI1 and VAV1 can cooperate in the regulation of the 8xGLI reporter. We showed that there is a significant increase in GLI transcriptional activity upon co-overexpression of GLI1 and VAV1 compared with the expression of only one of these constructs in both MIA PaCa2 and PANC1 cells (Fig. 2, *B* and *C*, *top*). Western blotting indicates equivalent protein expression of VAV1 and GLI1 when expressed on their own or coexpressed (Fig. 2, *B* and *C*, bottom). Furthermore, luciferase assays performed after knockdown of VAV1 in Panc 04.03 cells, followed by overexpression of GLI1, show a significant reduction in 8xGLI reporter activity (Fig. 2*D*). Taken together, these data indicate that VAV1 is a positive regulator of GLI1 transcriptional activity in PDAC cells.

### Nuclear localization of VAV1 leads to increased GLI activity

**Figure 3:**
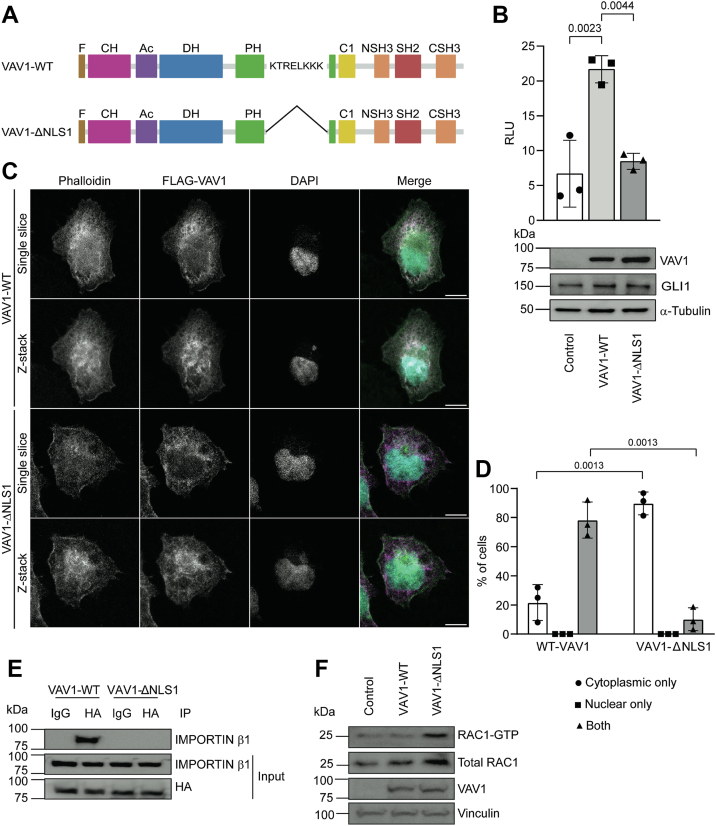
**Nuclear VAV1 required for GLI activation**. *A*, schematic of VAV1-WT (*top*) and VAV1-ΔNLS1 mutant (*bottom*). *B*, luciferase assay indicating relative GLI activation following transfection of the indicated VAV1 constructs in PANC1 cells (*top*; n = 3). One-way ANOVA was performed with a post hoc Tukey’s multiple comparison test. Representative Western blot image showing expression of the VAV1 constructs (*bottom panel*). *C*, single-slice and z-stack confocal microscopy images of immunofluorescence was performed in PANC1 cells transfected with VAV1-WT or VAV1-ΔNLS1 FLAG-tagged constructs (*magenta*): and counterstained with phalloidin (*green*) and DAPI (*cyan*) (n = 3; the scale bar represents 10 μm). *D*, quantification of the cellular localization of VAV1 in PANC1 cells for the same constructs used in (*C*) (n = 3, minimum of 80 cells counted per condition per replicate). Unpaired, two-tailed *t* tests were performed to compare the cytoplasmic only and both categories between the VAV1-WT and VAV1-ΔNLS1 samples. *E*, representative Western blot images after the coimmunoprecipitation assay between VAV1-WT or VAV1-ΔNLS1 and endogenous IMPORTIN in PANC1 cells (n = 3). *F*, representative Western blot images after RAC1-GTP pull-down (n = 3) were performed in PANC1 cells overexpressing VAV1-WT, VAV1-ΔNLS1, or empty vector (control). All data points indicate the average value for independent biological replicates. Ac, acidic domain AA 132 to 176 (inhibits DH, Tyr174 phosphorylation removes the inhibition); C1, atypical C1 domain AA 516 to 565; CH, calponin homology domain AA 1 to 119 (actin binding); DAPI, 4′,6-diamidino-2-phenylindole; DH, Dbl homology domain AA 185 to 375 (regulates GEF-dependent functions, activates GTPases); F, N-terminal FLAG tag; PH, Pleckstrin homology domain AA 398 to 508 (lipid and protein binding); SH2, Src homology 2 domain AA 671 to 765 (binds phosphotyrosine residues of other proteins); SH3, Src homology 3 domain AA 592 to 660/782 to 842 (binds proline-rich regions of other proteins).

Given that VAV1 can localize to the nucleus, we wanted to determine if nuclear localization was required for VAV1 to increase the activity of GLI transcription factors. To do this, we deleted amino acids 487 to 494 of VAV1, which are the amino acids associated with the first and only functional nuclear localization signal (NLS) present in VAV1 (VAV1-ΔNLS1, Fig. 3*A*) as previous studies have shown this deletion is sufficient to keep VAV1 from translocating to the nucleus (4). Interestingly, the activation of GLI factors is significantly reduced following overexpression of the VAV1-ΔNLS1 construct in PANC1 cells as compared with the VAV1-WT construct (Fig. 3*B*, *top*). Importantly, this was not because of a difference in protein levels, as Western blotting indicated similar overall expression between WT and ΔNLS1 VAV1 constructs (Fig. 3*B*, *bottom*). RT–qPCR analysis showed that relative expression of *GLI1*, *GLI2*, and *GLI3* was again unchanged *via* the overexpression of WT or ΔNLS1 VAV1 constructs (Fig. S2*B*). We confirmed *via* IF that the deletion of the NLS was sufficient to retain VAV1 in the cytoplasm of PANC1 cells (Fig. 3, *C* and *D*). Further analysis of the functionality of VAV1 nuclear localization confirmed that VAV1-WT coimmunoprecipitated (co-IP) with IMPORTIN β1, an importin that plays a central role as an active transport receptor with direct contact with the nuclear pore complex for cargo shuttling (29). Deletion of the NLS in VAV1 impairs the interaction with IMPORTIN β1 (Fig. 3*E*). Last, to evaluate if this deletion affects VAV1 GEF function, downstream RAC1 signaling was determined by showing GTP-bound RAC1 in PANC1 cells expressing WT or ΔNLS1 VAV1. Results show that VAV1-ΔNLS1 was able to activate GTP-bound RAC1 comparable to VAV1-WT (Fig. 3*F*). Altogether, these data support the functionality of nuclear VAV1 and indicate a necessity for its nuclear localization to modulate the activity of GLI transcription factors in PDAC cells.

**Figure 4:**
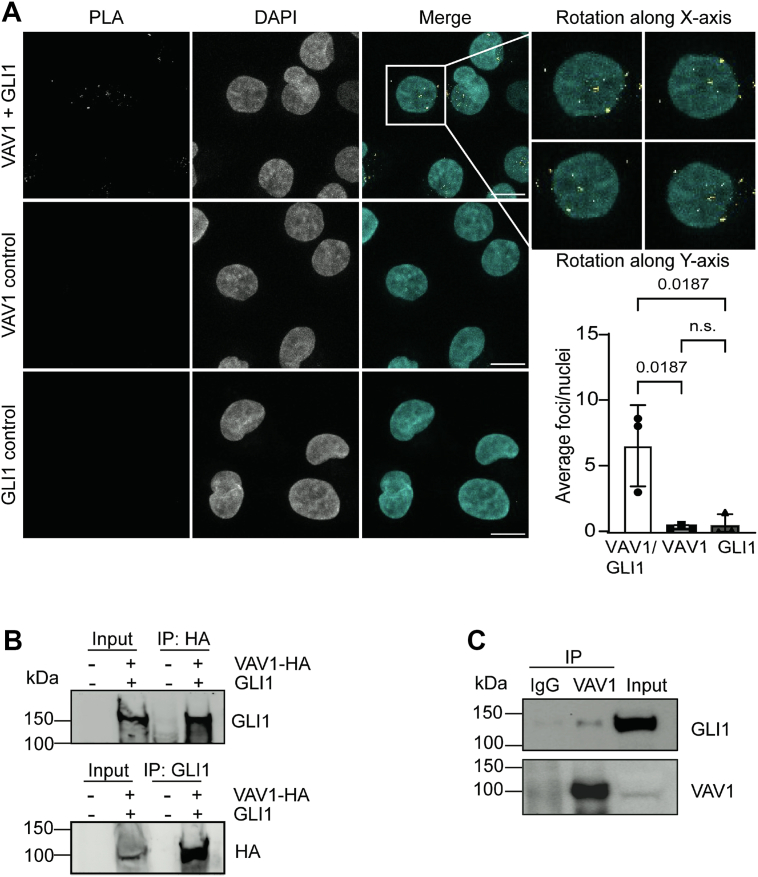
**VAV1 and GLI1 interact in the nucleus**. *A*, confocal images of a proximity ligation assay (PLA) were performed for VAV1 and GLI1, VAV1 alone, and GLI1 alone, in MIA PaCa2 cells (n = 3) (the scale bar represents 10 μm). *Bottom right panel*, quantification of foci per nuclei *via* 3D object counting using Fiji (ImageJ) software (n = 3, minimum 50 nuclei/assay). All data points indicate the average value for independent biological replicates. One-way ANOVA was performed with a post hoc Tukey’s multiple comparison test. *B*, representative Western blot images after co-IP assay in PANC1 cells, transfected with VAV1-WT HA-tagged and GLI1 (n = 3). *C*, representative Western blot images after endogenous co-IP assay in Capan-2 cells (n = 3). Co-IP, coimmunoprecipitation; HA, hemagglutinin.

Furthermore, to explore the nature of this GLI1–VAV1 interplay, we performed IF in transfected PANC1 cells with mCherry-VAV1 and hemagglutinin (HA)-GLI1. IF quantification using line scan analysis showed significant overlapping signal in the nucleus, suggesting a potential interaction between these molecules (Fig. S3, *A*–*B*). Using a proximity ligation assay (PLA) in MIA PaCa2 cells, we confirmed the endogenous interaction of VAV1 and GLI1 in the nucleus, with a small number of foci near the nuclear periphery (Fig. 4*A*). To determine the specificity of the interaction, a negative control PLA was performed with VAV1 and GAPDH, a nuclear protein not expected to interact with VAV1, which exhibited no positive PLA foci (Fig. S4). To further validate the interaction, co-IP was performed in PANC1 cells transfected with HA-tagged VAV1-WT and FLAG-tagged GLI1. Western blot analysis confirmed the interaction between VAV1 and GLI1 (Fig. 4*B*). Last, endogenous co-IP in VAV1-positive Capan-2 cells also demonstrated the interaction between VAV1 and GLI1 (Fig. 4*C*). These results suggest that VAV1 exerts a positive regulatory effect on GLI1 through a physical interaction occurring in the nucleus.

### VAV1 is required for the binding of GLI1 and its coactivator PCAF

**Figure 5:**
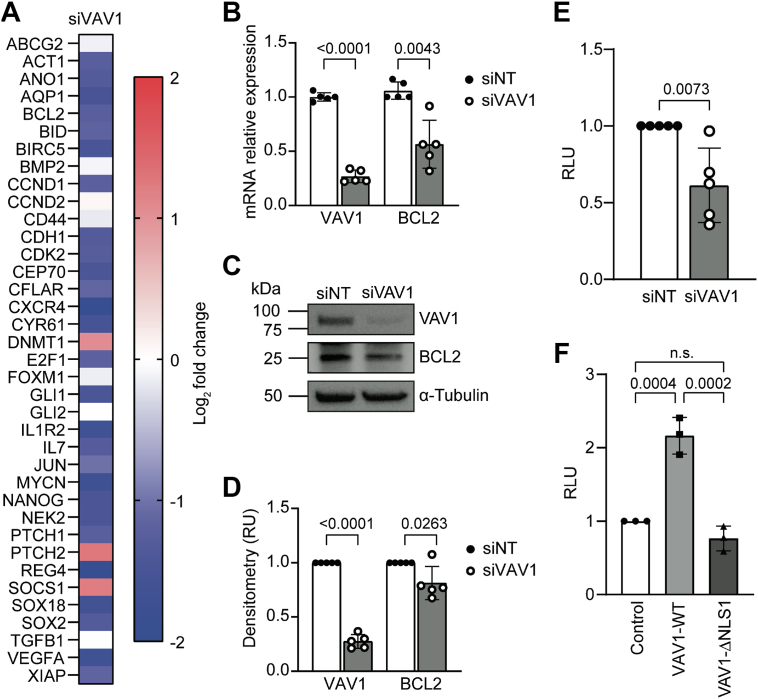
**VAV1 affects expression of GLI1 target genes and interacts with GLI-binding sites in the *BCL2* promoter**. *A*, heatmap showing expression changes of known GLI1 target genes following VAV1 knockdown *via* siRNA in MIA PaCa2 cells, average values from an n = 2. *B*, relative mRNA expression of VAV1 and BCL2 following siRNA knockdown of VAV1 in Panc 04.03 cells (n = 5). Unpaired, two-tailed *t* tests were performed to determine the statistical significance. *C*, representative Western blot image showing protein expression of VAV1 and BCL2 following siRNA knockdown of VAV1 in Panc 04.03 cells (n = 5). *D*, quantitation of Western blot bands from (*C*) *via* densitometric analysis using Fiji (ImageJ) software. Unpaired, two-tailed *t* tests were performed to compare siVAV1 sample to siNT sample. *E*, luciferase assay was performed in Panc 04.03 cells using a *BCL2* promoter reporter plasmid following siRNA knockdown of VAV1. Unpaired, two-tailed *t* tests were used to determine the statistical significance. *F*, luciferase assay was performed in PANC1 cells using a *BCL2* promoter reporter plasmid and the listed VAV1 constructs. One-way ANOVA was performed with a post hoc Tukey’s multiple comparison test. All data points indicate the average value for independent biological replicates.

To further elucidate the effects of VAV1 on GLI1 activity, we performed RT–qPCR of GLI1 target genes (30) following siRNA-based knockdown of VAV1 in MIA PaCa2 cells, which showed a reduction in the expression of a subset of GLI1 target genes (Fig. 5*A*). Given that both GLI1 and VAV1 have been shown to regulate BCL2 levels (VAV1 (31); GLI1 (32)), we chose this molecule as a model to study the interplay between VAV1 and GLI1. We first validate the reduction of *BCL2* mRNA expression in Panc 04.03 cells following knockdown of VAV1 (Fig. 5*B*). We also show a reduction in BCL2 protein levels following VAV1 knockdown in these PDAC cells (Fig. 5, *C* and *D*). Similar results were obtained using two additional VAV1 siRNAs (Fig. S5, *A*–*C*). To further investigate this regulatory mechanism of VAV1 on BCL2, we performed a luciferase assay using a *BCL2* promoter reporter following knockdown of VAV1 and found a reduction in *BCL2* promoter activity (Fig. 5*E*). Conversely, overexpression of VAV1-WT leads to increased *BCL2* promoter activity, whereas VAV1-ΔNLS1 fails to induce the activity of this promoter (Fig. 5*F*).

**Figure 6:**
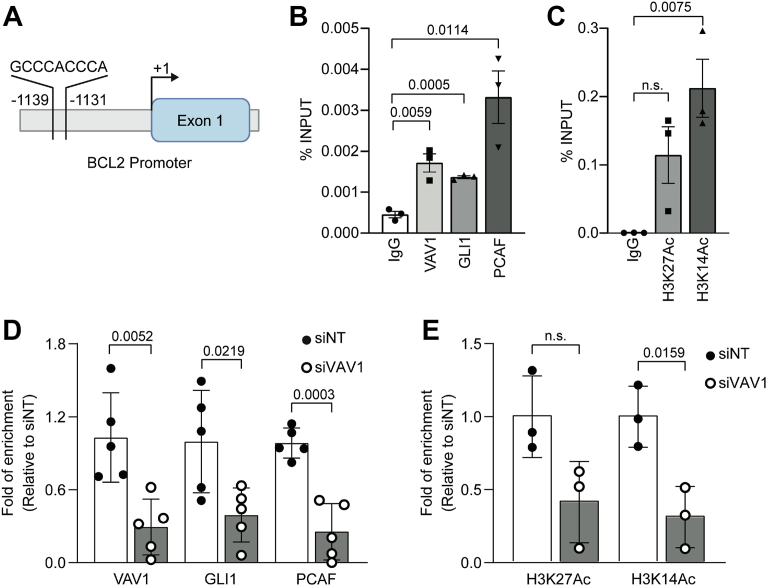
**VAV1 is necessary for GLI1 and PCAF binding at the *BCL2* promoter**. *A*, diagram of the GLI binding site present in the *BCL2* promoter sequence. *B*, basal ChIP–qPCR was performed for VAV1, GLI1, and PCAF at the GLI1 binding site in the *BCL2* promoter region in Panc 04.03 cells (n = 3). Unpaired, two-tailed *t* tests were used to compare each protein with their IgG control group. *C*, basal ChIP–qPCR was performed for H3K27Ac and H3K14Ac at the GLI1 binding site in the *BCL2* promoter region in Panc 04.03 cells (n = 3). Unpaired, two-tailed *t* tests were used to compare each protein with their IgG control group. *D*, normalized fold enrichments of ChIP–PCRs were performed for VAV1, GLI1, and PCAF at the GLI1 binding site in the *BCL2* promoter region following siRNA knockdown of VAV1 in Panc 04.03 cells (n = 5). Unpaired, two-tailed *t* tests were used to compare the siVAV1 group with the siNT group. *E*, normalized fold enrichments of ChIP–PCRs were performed for H3K27Ac and H3K14Ac at the GLI1 binding site in the *BCL2* promoter region following siRNA knockdown of VAV1 in Panc 04.03 cells (n = 3). Unpaired, two-tailed *t* tests were used to compare the siVAV1 group to the siNT group. All data points indicate the average value for independent biological replicates. ChIP, chromatin immunoprecipitation; qPCR, quantitative PCR.

Next, we sought to further define the binding of these molecules, along with the known GLI1 coactivator, PCAF (32), at a GLI binding motif present within the *BCL2* promoter region (Fig. 6*A*). VAV1, GLI1, and PCAF were found to bind to this region in Panc 04.03 cells (Fig. 6*B*). Analysis of two histone marks associated with active transcription (33) and GLI1 activity showed a statistically significant enrichment of H3K14Ac and a modest yet not statistically significant enrichment of H3K27Ac (Fig. 6*C*). Interestingly, knockdown of VAV1 significantly reduced the binding of GLI1 and PCAF and enrichment of H3K14Ac at this same GLI binding motif, without significantly affecting H3K27Ac at this region (Fig. 6, *D* and *E*). These data indicate a functional role for VAV1 involvement in the binding of GLI1 and associated coactivator to the promoter of *BCL2*, a known GLI1 target gene (32).

## Discussion

In this study, we have provided evidence of a novel mechanism underlying the pro-tumoral function of VAV1 in PDAC cells. Most mechanistic studies have looked at the cytoplasmic role of VAV1, which includes GEF-dependent functions, such as cytoskeletal remodeling (1, 12, 13), and GEF-independent functions, which includes controlling calcium flux (5). Only a few reports have examined the functionality of VAV1 outside this cellular compartment; for instance, Houlard *et al*. (4) showed a role for VAV1 in NFAT transcriptional dynamics by controlling NFAT shuttling into the nucleus in rat basophilic leukemia cells upon FcεRI stimulation. These authors (4) showed a reduction in NFAT transcription of *IL2* following expression of a VAV1 mutant that primarily localizes to the nucleus in Jurkat T-cells, indicating the necessity of VAV1’s cytoplasmic localization and subsequent nuclear translocation as a protein complex with NFAT to promote NFAT activity in these cells. Ksionda *et al*. (34) investigated the domains involved in VAV1 localization to the immunological synapse following stimulation of T-cells with antigen-presenting cells. In their work, disruption of the NLS in VAV1 was sufficient to keep VAV1 in the cytoplasm of T-cells, whereas disruption of the C-terminal SH3 domain led to increased nuclear localization in T-cells, recapitulating Houlard *et al*. results. Both studies utilized some form of external stimulation before seeing the presence of wild-type VAV1 in the nuclei of their model cells, whereas we show the constitutive presence of VAV1 in the nuclei of PDAC cells with no exogenous stimulation required. We also demonstrated that in the absence of any known RAC1 stimuli the ΔNLS VAV1 showed no difference in the activation of RAC1 compared to the WT. Alongside these data, our study indicates that overexpression of VAV1 in PDAC cells leads to increased activity of GLI transcription factors, which has not been previously shown. Using IP and PLA assays we define the endogenous interactions between VAV1 and GLI1 in PDAC cells and using ChIP assays we demonstrate the requirement of VAV1 for GLI1 and PCAF binding at the GLI binding motif in the BCL2 promoter region, as GLI1 and PCAF are no longer present when VAV1 is knocked down. We also show nuclear VAV1, specifically, is required for VAV1 to exert a positive regulatory effect on GLI transcriptional activation in PDAC, which contrasts with the model of VAV1 regulation on NFAT transcriptional dynamics as presented by Houlard *et al*. This indicates that there are various mechanisms by which VAV1 can alter transcriptional dynamics that is specific to each transcription factor. Thus, our study determined a functional role for nuclear VAV1 in PDAC cells through increasing GLI transcription factor activity.

Canonical GLI activation occurs downstream of Hh signaling, where GLI transcription factors are required for organ development and maintenance of pluripotent and somatic stem cells in a variety of tissues (35, 36, 37, 38, 39, 40, 41). Canonical activation of GLI factors by Hh signaling takes place in the primary cilium and involves the binding of Hh ligands to the 12-pass transmembrane receptor Patched 1 (PTCH1), relieving the repression of PTCH1 on the 7-pass transmembrane receptor Smoothened (40, 42, 43, 44, 45, 46). This leads to the release of full-length GLI from Suppressor of Fused (SUFU), allowing GLI to translocate to the nucleus and activate transcription of GLI target genes (47, 48, 49, 50). GLI can also be modulated in the absence of Hh ligands. Typically, this non-canonical regulation involves its activation independent of the Hh ligand–receptor complex (51). Some of these non-canonical pathways include mitogen-activated protein kinase signaling, which has been found to increase GLI transcriptional activity and nuclear localization, as well as promote GLI protein stability in cancers from a variety of tissues, including pancreatic tumors (15, 52, 53, 54, 55, 56, 57). GLI transcriptional activity, protein stability, and nuclear localization can also be increased downstream of PI3K–AKT signaling (52, 55, 57, 58, 59, 60). Some studies have indicated that PKC isoforms can have opposite effects on the activity of GLI transcription factors, such as PKCα reducing GLI transcriptional activity, whereas PKCδ increases GLI transcription in normal cells (61). The opposite is true in cancer cells in regard to the PKC isoforms (62). Recent studies have indicated that c-Jun, which is downstream of JNK signaling, can stabilize GLI2 (63). In this study, we demonstrate VAV1 is an additional non-canonical regulator of GLI transcriptional activity in PDAC cells. VAV1 has also been shown to be involved in regulating mitogen-activated protein kinase (3, 6, 64), PI3K–AKT (65, 66), and JNK signaling (1, 2, 67), as well as regulating PKCθ signaling (64, 67, 68). Given the extensive overlap in signaling pathways regulated by VAV1 and GLI1 future studies should aim at determining the molecules mediating the crosstalk between them.

In our study, overexpressing VAV1 led to increased activity of GLI transcription factors. We chose to focus on GLI1 as it only functions as a transcriptional activator. Our luciferase data indicate an additive effect of GLI1 and VAV1 on GLI1 transcriptional activity in PDAC cells, which has not been previously shown. Our data indicate an interaction between VAV1 and GLI1 occurring in the nucleus of PDAC cells, where VAV1 exerts a regulatory effect on GLI1 binding at the GLI1 target gene, *BCL2*. We determined that VAV1 is necessary for the binding of GLI1 in this region as the reduction of VAV1 *via* siRNA removed GLI1 binding at the *BCL2* promoter. To further understand the mechanism(s) underlying VAV1 regulated gene transcription, additional studies would be necessary to define if GLI1 and VAV1 interaction is direct and the domains that are mediating it. Overall, this work expands on the functional role of nuclear VAV1 and further indicates how the subcellular localization of VAV1 affects its functional output. Ultimately, we discovered a mechanism for nuclear VAV1 in PDAC, where VAV1 exerts regulatory effects on GLI1 transcriptional activity and DNA binding. This novel functional interaction of VAV1 and GLI1 could provide a new therapeutic route for PDAC patients.

## Experimental procedures

### Cell lines and culture conditions

PANC1 (catalog no.: CRL-1469), Panc 04.03 (catalog no.: CRL-2555), Panc 05.04 (catalog no.: CRL-2557), MIA PaCa2 (catalog no.: CRL-1420), and Capan-2 (catalog no.: HTB-80) PDAC cell lines were obtained from the American Type Culture Collection. PANC1 and MIA PaCa2 cells were cultured in Dulbecco's modified Eagle's medium (Corning; catalog no.: 10-013-CV) supplemented with 10% fetal bovine serum (FBS) (Corning; catalog no.: 35-010-CV). Panc 04.03 and Panc 05.04 cells were cultured in RPMI1640 media (Corning; catalog no.: 10-040-CV) supplemented with 15% FBS. Capan-2 cells were cultured in McCoy’s 5a media (Gibco; catalog no.: 16600-082) supplemented with 10% FBS.

### Plasmids and siRNA

The WT plasmid for VAV1 and pcDNA-FLAG-VAV1-WT was previously described (69). Sanger sequencing was performed to verify N-terminal in-frame FLAG tag and the sequence of VAV1 to be transcript variant 1 (NM_005428.4). Primers were designed and obtained from IDT to create amino acid 487 to 494 NLS deletion plasmid (pcDNA-FLAG-VAV1-Del NLS1). Fragments to remove NLS were amplified using the Terra Direct PCR Red Dye Premix kit (TakaRa; catalog no.: 639286) along with the following primers: GIB-VAV1-F1, 5′-CTGGCTCACTATGGCCGGCCCAA-3′/GIB-VAV1-NLS1-R1, 5′-CAAACTGCTCCATCCAGAAGAACAGCTCA-3′ (310 bp) and GIB-VAV1-NLS1-F1, 5′-TATGAGCTGTTCTTCTGGATGGAGCAGTTTG-3′/GIB-VAV1-R1, 5′-TATCATGTCTGCTCGAAGCGGCCGCAG-3′ (1125 bp). WT VAV1 plasmid was opened with restriction enzymes FseI and NotI, and the vector backbone (6848 bp) was gel purified along with PCR fragments for NLS1 deletion. The Gibson Assembly Cloning Kit (NEB; catalog no.: E5510S) protocol was followed according to the manufacturer to create pcDNA-FLAG-VAV1-ΔNLS1 plasmid. Sanger sequencing was performed to confirm plasmids and proper deletion of NLS. Gibson assembly was again utilized to move VAV1-WT into the pcDNA3.1-HA vector. VAV1-WT was amplified out of the pcDNA-FLAG-VAV1-WT plasmid with primers GIB-VAV1-HA-F1 5′-TATAGGGAGACCCAAGCTTATGGAGCTGTGGCGCCAATGC-3′/GIB-VAV1-HA-R1 5′-AGCTCCTGGCGCGGATCCGCAGTATTCAGAATAATCTTCCTCCAC-3′ (2572 bp). The vector backbone (pcDNA-HA) was opened using restriction enzymes HindIII and BamHI, and the VAV1-WT fragment was ligated using the Gibson assembly cloning kit protocol. Sanger sequencing confirmed the pcDNA-VAV1-WT-HA plasmid. The pmCherry-C1-VAV1-WT plasmid was kindly gifted by Dr Gina Razidlo (Mayo Clinic). Sanger sequencing was performed to verify N-terminal in-frame mCherry and WT VAV1 sequence to be transcript variant 1 (NM_005428.4). The HA-GLI1 plasmid was kindly gifted by Dr Cheng (Igen International). Full-length human GLI1 was cloned into the pCMV14-3xFLAG vector (Sigma–Aldrich) to generate GLI1-FLAG (Nye, 2014; PMID: 24739390). The NFAT luciferase reporter plasmid was a kind gift from Dr David McKean (70) (Mayo Clinic), the NF- κB reporter plasmid was a gift from Dr Carlos Paya (71) (Mayo Clinic), and the 8xGLI luciferase reporter plasmid was a kind gift from Dr Chi-Chung Hui (72) (University of Toronto). The 6X-STAT-synthetic luciferase (73) and the M50 Super 8X TOPFlash (74) reporter plasmids were purchased from Addgene (6X-STAT—Research Resource Identifier: Addgene_37392; 8X TOPFlash—Research Resource Identifier: Addgene_12456). *BCL2* promoter luciferase construct was a gift from Dr Boxer (Center of Molecular Biology in Medicine, Veterans Affairs Palo Alto Health Care System).

Individual Qiagen siRNAs were obtained for VAV1 as Hs_VAV1_2 FlexiTube siRNA (catalog no.: 1027418, GeneGlobe ID: SI00077007) and Hs_VAV1_3 FlexiTube siRNA (catalog no.: 1027418, GeneGlobe ID: SI00077014), as well as an AllStars Negative Control siRNA (catalog no.: 1027281). Individual Dharmacon ONTARGETplus siRNAs were obtained for VAV1 (catalog no.: J-003935-09-0020; sequence—UCAAAUACAAGGAGAGGUU) and nontargeting control siRNA (control 1, catalog no.: D-001810-01-20; sequence—UGGUUUACAUGUCGACUAA). Lyophilized siRNA was reconstituted to a final concentration of 20 μM.

### Transfections

DNA transfections were performed using X-tremeGENE HP DNA Transfection Reagent (Roche, catalog no.: 6366244001) according to the manufacturer’s protocol for a 2:1 ratio of X-tremeGENE reagent to micrograms of DNA. The total DNA quantity used for cotransfections was identical across all conditions within each experiment. RNA transfections were performed using Lipofectamine RNAiMAX Transfection Reagent (Invitrogen, catalog no.: 13778500) according to the manufacturer’s protocol. Transfections occurred 24 h after plating. MIA PaCa2 and PANC1 cells were plated at a density of 1.5 × 10^6^ cells on 10-cm dishes for nuclear fractionation studies or at 2.5 × 10^4^ cells per well in a 24-well dish for luciferase assays. For IF studies, PANC1 cells were plated in triplicate at 5.0 × 10^4^ cells on UV-sterilized coverslips in 12-well dishes. Panc 04.03 cells were plated at a density of 2.5 × 10^6^ cells on 10-cm and 15-cm dishes for luciferase and ChIP studies, respectively. For Panc 04.03 cells used in luciferase assays, siRNA-transfected cells were replated after 24 h at a density of 4.0 × 10^4^ cells per well in a 24-well dish, maintaining a 10-cm dish to quantify the siRNA knockdown efficiency. Cells were harvested and processed 24 h following DNA transfections or 72 h following siRNA transfections. For RAC1 activity assays, PANC1 cells were seeded at a density of 1.0 × 10^6^ cells per 10-cm dish. Twenty-four hours post-transfection, FBS was removed, and cells were harvested following an additional 24-h serum starvation period. For immunoprecipitation (IP) assays, PANC1 cells were seeded at a density of 1.0 × 10^6^ cells per 10-cm dish and collected 24 h after transfection. To ensure comparable protein expression levels, the amount of plasmid DNA was adjusted accordingly, with EV added to equalize the total DNA across samples.

### Nuclear fractionation

Nuclear fractionation was performed following the REAP method (75). Cells were washed in 1x PBS and then scraped into 1 ml of PBS, transferred to 1.5 ml microcentrifuge tubes, and centrifuged using a 10 s “pop-spin” in a table-top centrifuge. The supernatant was removed, and 900 μl of 0.1% NP-40 in PBS was added to samples and triturated five times using a P1000 pipette. Three hundred microliters was removed as “whole cell lysate,” and 100 μl of 4x Laemmli buffer was added to this fraction, followed by keeping this fraction on ice until the sonication step. The remaining lysates were centrifuged for a 10 s pop-spin. Three hundred microliters of the remaining lysate were removed as labeled “Cytosolic fraction,” and 100 μl Laemmli buffer was added to this fraction, followed by boiling for 1 min. The remaining supernatant was removed from the pellet. The pellet was resuspended in 1 ml of 0.1% NP-40 in PBS and centrifuged for a 10 s pop-spin, and the supernatant was removed. The pellet (∼20 μl) was resuspended in 180 μl of 1x Laemmli buffer and labeled “Nuclear fraction.” The whole-cell lysate and nuclear fractions were sonicated at power level 3, two times for 5 s each, followed by boiling these fractions for 1 min. Thirty microliters, 30 μl, and 15 μl of the whole-cell lysate, cytosolic fraction, and nuclear fraction, respectively, were loaded for SDS-PAGE. H3 and α-tubulin were used as nuclear and cytosolic controls, respectively. See the Western blotting section for further details.

### Cell starvation

PANC1 cells transfected with WT VAV1 were utilized for cell starvation analysis. One day post transfections, cells were depleted and grown in media containing 0.5% FBS for 24 or 48 h, whereas one group was grown normally in complete media. At 24 h post serum depletion, cells were either collected, replenished with serum, or remained starved for a total of 48 h. The remaining cells were collected 48 h post initial starvation. Nuclear fractionation (see above) was performed on each condition, and VAV1 localization was assessed *via* Western blot. Relative VAV1 expression in cytoplasmic and nuclear compartments was calculated using densitometry relative to the compartmental controls α-tubulin and H3, respectively.

### Western blotting

Cells were lysed using radioimmunoprecipitation assay buffer, supplemented with 1 mM PMSF and cOmplete Protease inhibitor (1 tab per 50 ml of radioimmunoprecipitation assay; Roche, catalog no.: 11836145001), followed by sonication using three cycles of 5 s on followed by 5 s off at a power setting of 3. The lysates were centrifuged at maximum RPMs for 15 min to pellet the cell debris. Lysates were quantified using Pierce BCA kit reagents (ThermoFisher, catalog no.: 23227) following the manufacturer's protocol. Lysates were combined with SDS loading buffer and DTT and incubated at 95 °C for 5 min. Proteins were separated by SDS-PAGE (4–15%) and transferred to a polyvinylidene difluoride membrane. Membranes were blocked for 1 h at room temperature in 5% nonfat milk in 1x Tris-buffered saline containing 0.1% Tween-20. Membranes were incubated overnight at 4 °C with the following primary antibodies in blocking buffer: VAV1 (1:1000 dilution; [Panc 04.03 and Panc 05.04] Cell Signaling, catalog no.: 4657S; [Capan-2, MIA PaCa2, and PANC1] Invitrogen, catalog no.: MA5-31488), FLAG (1:1000 dilution; Novus, catalog no.: NBP1-06712SS), GLI1 (1:1000 dilution; Cell Signaling, catalog no.: 3538S), BCL2 (1:500 dilution; BD Biosciences, catalog no.: 610538), HA (1:1000 dilution; Cell Signaling, catalog no.: 2367S), GAPDH (1:2000 dilution; Cell Signaling, catalog no.: 2118S), H3 (1:1000 dilution; Cell Signaling, catalog no.: 3638S), α-tubulin (1:2000 dilution; MilliporeSigma, catalog no.: T9026). Membranes were washed with Tris-buffered saline with Tween-20 three times for 10 min, followed by a 1-h incubation with either an anti-rabbit (1:5000 dilution), anti-rat (1:5000 dilution), or anti-mouse (1:3500 dilution) immunoglobulin G (IgG) secondary antibody, conjugated to horseradish peroxidase (MilliporeSigma, catalog no.: AP132P [rabbit], AP136P [rat], and AP124P [mouse]). Membranes were washed another three times for 10 min, followed by developing blots using SuperSignal West Pico PLUS Chemiluminescent Substrate (ThermoFisher, catalog no.: 34580). Imaging was performed using a Bio-Rad ChemiDoc MP Imaging System (catalog no.: 12003154). Where performed, densitometric analysis was done with Fiji (ImageJ) software (76), using loading control bands for normalization of samples.

### Luciferase reporter assay

One day post-transfection, samples were lysed at room temperature for 15 min using a passive lysis buffer (Promega, catalog no.: E194A), followed by reading samples for luciferase concentration using a Berthold luminometer (Berthold Technologies; Centro XS^3^ LB 960) and Luciferase Assay System (Promega, catalog no.: E1501). Total protein was quantified using the Bio-Rad Protein Assay (catalog no.: 5000006) according to the manufacturer's protocol. Luciferase signal was normalized to total protein to account for any variation among samples and then normalized to their respective experimental control. To avoid potential oversaturation of activity when cotransfecting VAV1 with GLI1, a direct transcriptional activator of 8xGLI, the amount of VAV1 used in the experiments shown in Figure 2*B* was reduced to one-fourth of that used in Figure 2*A*.

### Proximity ligation assay

MIA PaCa2 cells were plated on UV-sterilized coverslips 1 day prior to performing the PLA. Cells were fixed at room temperature using 3.6% paraformaldehyde, following by permeabilizing the cells on ice using 0.1% Triton X-100. After permeabilization, proximity ligation was performed according to the Duolink PLA Fluorescence Protocol, using green detection reagents (MilliporeSigma, catalog no.: 92014), and the following antibodies: mouse anti-VAV1 (Invitrogen, catalog no.: MA5-31488, 1:250 dilution) and rabbit anti-GLI1 (Novus, catalog no.: NBP2-68877, 1:500 dilution) or rabbit anti-GAPDH (Cell Signaling, catalog no.: 2118S, 1:200 dilution). Amplification time used for the VAV1/GLI1 antibodies was 180 min and for VAV1/GAPDH antibodies was 60 min. Images were taken on a Zeiss LSM 800 confocal microscope using Airyscan and processed using Fiji (ImageJ) software (76).

### Immunofluorescence

One day post-transfection, cells were fixed using a Pipes-based fixative (0.1 M Pipes, 1 mM EGTA, 3 mM MgSO_4_, and 2.5% formaldehyde) for 20 min at room temperature, followed by washing three times in Dulbecco’s PBS (DPBS). Cells were permeabilized in 0.1% Triton X-100 for 5 min on ice, followed by washing three times in DPBS (for mCherry transfections, continue to 4′,6-diamidino-2-phenylindole [DAPI] staining). Coverslips were blocked for 60 min at 37 °C in a humidified chamber using blocking buffer (0.3 M glycine, 5% glycerol, and 5% normal goat serum in 1x PBS). Coverslips were incubated with primary antibodies diluted in blocking buffer (rat anti-FLAG, 1:100 dilution; Novus, catalog no.: NBP1-06712SS) for 2 h at 37 °C in a humidified chamber. One coverslip for each condition had no primary antibody present as a control coverslip. Coverslips were washed 3 × 10 min with DPBS, followed by incubation with the secondary antibody (goat anti-rat AF 594, 1:500 dilution; Invitrogen, catalog no.: A11007) diluted in blocking buffer for 1 h at 37 °C in a humidified chamber. Coverslips were washed 3 × 10 min with DPBS. For localization comparison of WT VAV1-WT-FLAG and VAV1-ΔNLS-FLAG coverslips were incubated for 15 min in Phalloidin (Alexa Fluor 647, 1:100 dilution; Cell Signaling, catalog no.: 8940S), followed by 2 5-min washes with DPBS. Last, coverslips were incubated with a 300 nM DAPI solution to stain nuclei for 3 min, followed by 2 5-min washes in DPBS. Coverslips were mounted using ProLong Diamond Antifade Mountant (Invitrogen, catalog no.: P36961). Coverslips were allowed to cover 24 h before imaging on a Zeiss LSM 800 confocal microscope using Airyscan. Z-stacks were obtained by imaging at 0.5 μm increments through a single cell and portrayed as a maximum projection. Images were processed using Fiji (ImageJ) software (76).

### Line scan analysis

PANC1 cells transfected with FLAG-VAV1-WT or mCherry-VAV1-WT and IF performed were used in line scan analysis. Fiji (ImageJ) software (75) was utilized in processing the middle *z*-plane of Z-stack confocal images, with a minimum of 30 cells analyzed per treatment. For analysis, a measurement line was drawn through the long axis of each nucleus to generate signal profile plots. Each measurement was normalized to a distance of 100 to account for differences in nuclear size. The intensity of VAV1 across the cells was then divided by the maximum signal along the line to show normalized relative intensity of VAV1 or GLI1 compared with DAPI.

### Chromatin immunoprecipitation

DNA/protein crosslinking was performed using a final concentration of 1% formaldehyde and quenched with glycine. When performed following siRNA transfections, crosslinking occurred 72 h post-transfection. Following lysis, chromatin was sheared by sonication (45 cycles of 30 s sonication followed by 30 s of rest; Diagenode Bioruptor 300). ChIP was performed as previously described (77) using either 2 μg of normal rabbit IgG (Cell Signaling, catalog no.: 2729S) or rabbit anti-GLI1 (Novus Biologicals, catalog no.: NB600-600) or 5 μg of normal rabbit IgG or a previously described rabbit polyclonal antiserum to VAV1 (78). Primers were used to amplify a GLI1 binding site within the *BCL2* promoter region (basal ChIP forward, GCTAGGGGCTATTCATGCTGATTA; basal ChIP reverse, GGGAAGGGGTTTATCAAGGGCTTT; siVAV1 ChIP forward, ACACACGTCTGCGAGTGTGAATGT; siVAV1 ChIP reverse, TCCCTCTGTCCCTAACACCTTT). qPCR was performed on the samples using SsoAdvanced Universal SYBR Green Supermix (Bio-Rad, catalog no.: 1725274). Amplification was performed using a Bio-Rad C100 Tough Thermal Cycler and a CFX384 Real-Time System with the following reaction conditions: 95 °C for 5 min, followed by 44 cycles of 95 °C for 30 s, 61 °C for 45 s, and 72 °C for 1 min. Normalization following qPCR was performed by subtracting the Log2 of the input dilution factor from the input Cq value, which was then subtracted from the Cq of the paired ChIP sample. The 2ΔCq method was then used to calculate the percent input.

### IP assay

All IP assays were performed as previously described (77). For endogenous IP, Capan-2 (4–5 × 10^6^) cells were incubated with 2 μg of anti-VAV1 (Cell Signaling, catalog no.: 4657S) or normal rabbit IgG (Cell Signaling, catalog no.: 2729S). Western blotting of endogenous proteins was performed as previously described, using anti-GLI1 (Origene, catalog no.: TA8033969) and anti-VAV1 (Cell Signaling, catalog no.: 4657S). For the VAV1–GLI1 overexpression IP, PANC1 (2 × 10^6^) cells overexpressing either VAV1-HA–tagged or GLI1-3xFLAG were incubated with 2 μg of anti-GLI1 (Cell Signaling, catalog no.: 3538S) or 0.05 μg of anti-HA (Cell Signaling, catalog no.: 2367S). Transfected proteins were detected by Western blot using anti-HA and anti-GLI1 antibodies. For the VAV1–IMPORTIN IP experiments, 24 h after transfection, PANC1 (2 × 10^6^) cells overexpressing VAV1-WT or VAV1-ΔNLS1, both FLAG-tagged, were incubated with 0.05 μg of anti-HA or normal mouse IgG (Diagenode, catalog no.: C15400001). Western blotting for immunoprecipitated proteins was detected using anti-VAV1 (Invitrogen, catalog no.: MA5-31488) and anti-IMPORTIN β1 (Invitrogen, catalog no.: MA3-070).

### RAC1 activity assay

To evaluate the activation of RAC1, RAC1-GTP was recovered using the PAK-GST Protein Beads Pull-down Activation Assay kit (Cytoskeleton, catalog no.: PAK02). Forty-eight hours after transfection, PANC1 cells overexpressing VAV1 WT, ΔNLS1, or EV were lysed in cold GTPase activation buffer (50 mM Tris–HCl, pH 7.5, 5 mM MgCl_2_, 0.5% NP-40, 10% glycerol, 500 mM NaCl, 1 mM PMSF, 10 μg/ml leupeptin, 5 μg/ml aprotinin, 1 mM Na_3_VO_4_, and 1X PIC), vortexed and centrifuged for 10 min at 18,000*g* at 4 °C. Aliquots were taken from the supernatant to compare protein amounts. Lysates (750 μg) were incubated with 20 μg agarose-conjugated PAK-GST at 4 °C for 1 h. After incubation, the beads were washed two times in cold GTPase activation buffer, resuspended in 40 μl Laemmli sample buffer, and boiled for 5 min at 95 °C. Active RAC1 was detected by Western blot by using a RAC1 antibody (BD Biosciences, catalog no.: 610651).

### Reverse transcription and real-time qPCR

Total RNA was extracted from cells using TRIzol reagent (Invitrogen, catalog no.: 15596018) following manufacturer's protocol. Two micrograms of total RNA were reverse transcribed using a High-Capacity cDNA Reverse Transcription Kit (Applied Biosystems, catalog no.: 4368813). Real-time qPCR was performed to measure relative gene expression of human genes, using SsoAdvanced Universal SYBR Green Supermix and the following primer sets: VAV1, GLI1, GLI2, GLI3, BCL2, TBP, and HPRT (Table 1). Amplification was performed using a Bio-Rad C1000 Touch Thermal Cycler and a CFX384 Real-Time System with the following reaction conditions: 50 °C for 2 min, 95 °C for 10 min, followed by 40 cycles of 95 °C for 15 s, 60 °C for 1 min. Normalization of mRNA levels within the same samples was performed by using an equally weighted combined housekeeping value calculated from TBP and HPRT. The 2ΔCq method was used to calculate results.

**Table 1:** List of primers used for RT–qPCR studies for mRNA expression

Gene	Forward primer	Reverse primer
*VAV1*	CTGTATGACTGCGTGGAGAAT	CGCTTGTCATACTCTGTCATCT
*GLI1*	TTCCCAACTTGCCAGCTGAA	ACAGGGGATCCTGTATGCCT
*GLI2*	AGACGACGTGGTGCAGTACATCAA	CAGCTGCTGCATGTAGTTTACCCT
*GLI3*	GAAGTTCGGGGACTTGACAGC	TGTCCAGGACTTTCATCCTCATT
*BCL2*	GTGGATGACTGAGTACCTGAAC	GAGACAGCCAGGAGAAATCAA
*TBP*	TATAATCCCAAGCGGTTTGC	CCCAACTTCTGTACAACTCTAGCA
*HPRT*	CCTGGCGTCGTGATTAGTGAT	AGACGTTCAGTCCTGTCCATAA

### Custom GLI target qPCR plates

Custom 384-well plates were prepared by Bio-Rad-containing wells with lyophilized, predesigned SYBR Green primers against previously identified direct human gene targets of GLI1 or GLI2 (*ABCG2*, *ACT1*, *ANO1*, *AQP1*, *BCL2*, *BID*, *BIRC5*, *BMP2*, *CCND1*, *CCND2*, *CD44*, *CDH1*, *CDK2*, *CEP70*, *CFLAR*, *CXCR4*, *CYR61*, *DNMT1*, *E2F1*, *FOXM1*, *GLI2*, *IL1R2*, *IL7*, *JUN*, *MYCN*, *NANOG*, *NEK2*, *PTCH1*, *PTCH2*, *REG4*, *SOCS1*, *SOX18*, *SOX2*, *TGFB1*, *VEGFA*, and *XIAP*). MIA PaCa2 cells were transfected with individual Qiagen siRNAs against VAV1 or with NT siRNA as described above. After 3 days, RNA was extracted and cDNA was prepared as described above. A 10 μl reaction mixture of cDNA, SYBR Green (Bio-Rad), and water was added to each well, and qPCR was performed as above. Cq values and PCR melting curves for each primer pair were examined, and only primer pairs that showed significant amplification and amplified single products were included in further analysis. Reactions were run concurrently for expression of GLI1, GLI2, and housekeeping genes (TBP and GAPDH) in the same cDNA samples using conventional real-time PCR as described above. Relative mRNA expression was calculated by normalization to GAPDH and TBP controls. Biological duplicates of each siRNA treatment were run using the premade plates, and the averages were calculated in Excel. Gene expression heatmaps were generated using the GraphPad Prism (version 10.3.1; GraphPad Software, Inc).

### IHC and cBioPortal analysis

IHC images for VAV1 staining in human pancreas tissues were obtained from the Human Protein Atlas (v24): www.proteinatlas.org (17). The images are available at the following URLs: (normal pancreas) https://images.proteinatlas.org/1864/163102_A_1_3.jpg; (PDAC) https://images.proteinatlas.org/1864/163101_B_4_5.jpg. Images were cropped, and scale bars were added using Fiji (ImageJ) software (76). Expression of VAV1 observed from Human Protein Atlas was further investigated using cBioPortal to determine the ectopic expression of VAV1 in PDAC occurs as the WT form of VAV1 and not a mutant version. Five PDAC cohorts (79, 80, 81, 82, 83) showed only eight total somatic mutations, five copy number amplifications, and one patient exhibiting a homodeletion in VAV1. Making the number of VAV1 alterations of mutations 1.1% across the five cohorts (989 patients).

### Statistical analysis

Statistical analysis was performed using GraphPad Prism software (version 10.3.1). Samples were tested for normality using the Shapiro–Wilk test, because of sample sizes, followed by either an unpaired, two-tailed *t* test for two-sample comparisons or an ordinary one-way ANOVA for three or more samples. Datasets analyzed by ANOVA were further analyzed with appropriate post hoc testing. When experimental groups were compared with a single control group, Dunnett’s multiple comparison test was performed. When each group was compared with every other group, Tukey’s multiple comparison test was performed. *p* Values are reported in each figure.

## Data availability

All data generated or analyzed during this study are included in this published article and its supplementary information files.

## Supporting information

This article contains supporting information.

## Conflict of interests

The authors declare that they have no conflicts of interest with the contents of this article.
