# Supplementary material for: Nuclear VAV1 increases GLI1-dependent transcription in pancreatic cancer cells
Source: J Biol Chem. 2025 Nov 26;302(1):110988. doi: 10.1016/j.jbc.2025.110988 (PMC12765065; doi:10.1016/j.jbc.2025.110988)
Supplement: Supplementary Material 1 [file mmc1.docx]

**
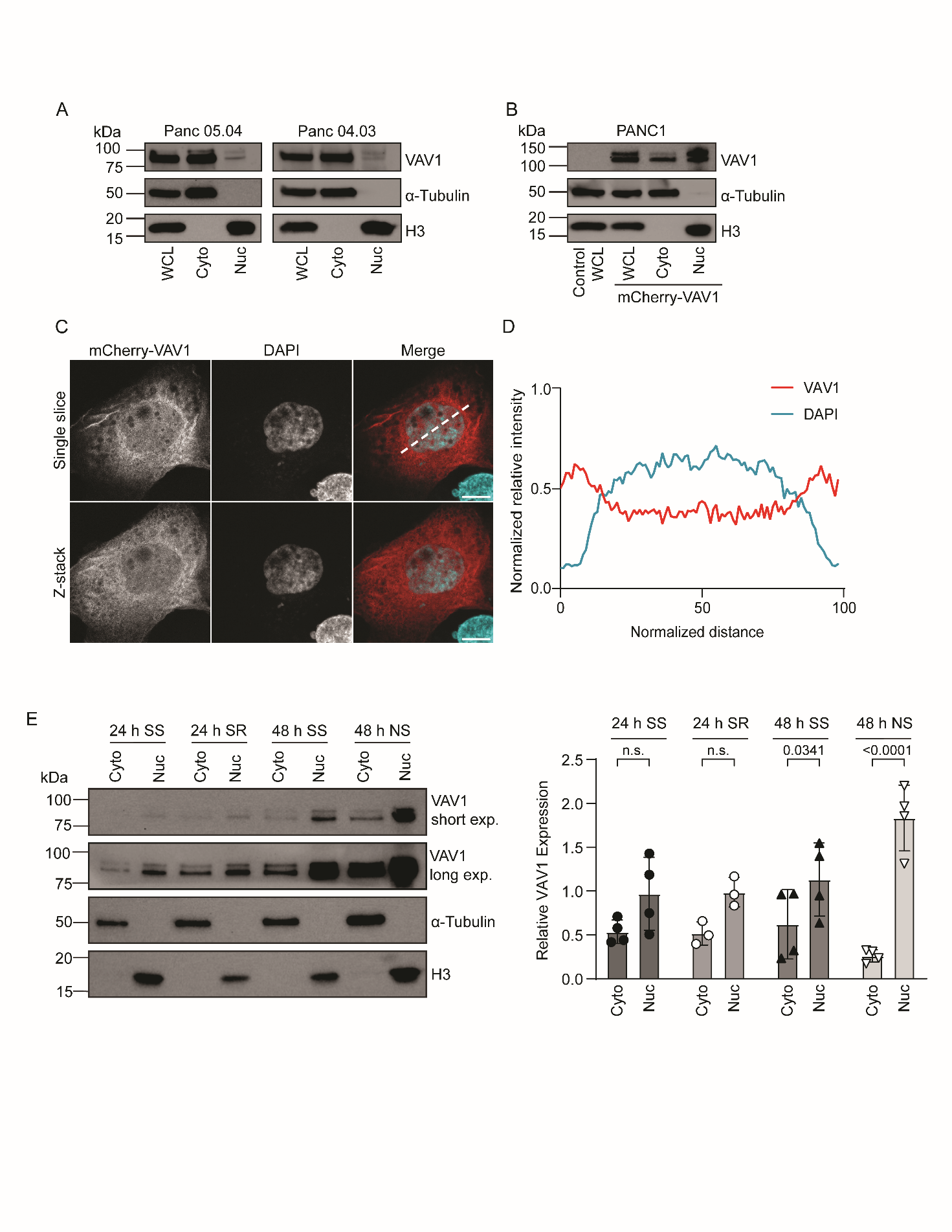
**

**Figure S1.** Dynamics of VAV1 localization in PDAC cells. (A) Representative Western blot images of a nuclear fractionation performed in Panc 05.04 (left; n=3) and Panc 04.03 (right; n=2) cells (WCL - whole cell lysate, Cyto - cytoplasmic, Nuc - nuclear). (B) Representative Western blot images of a nuclear fractionation performed in mCherry-VAV1 expressing PANC1 cells (n=2). (C) Representative single slice and z-stack of confocal microscopy image of mCherry in PANC1 cell expressing an mCherry-tagged wild-type VAV1 construct (Scale bar = 10 µm). The dashed line indicates where the line scan analysis was performed in (D). (D) Line scan analysis profile plot of mCherry-VAV1 and DAPI signal from (C) (n=30). (E) Representative Western blot images and densitometry quantification of nuclear fractionation assays performed in PANC1 cells transfected with FLAG-VAV1-WT. Twenty-four hours after transfection, cells were subjected to serum starvation (SS) 24-48h, 24h serum reinduced (SR), or cells that were never starved (NS) (n=3; Cyto - Cytoplasmic; Nuc - Nuclear). Two-way ANOVA was performed with Sidak’s multiple comparison test. All data points indicate the average value for independent biological replicates.


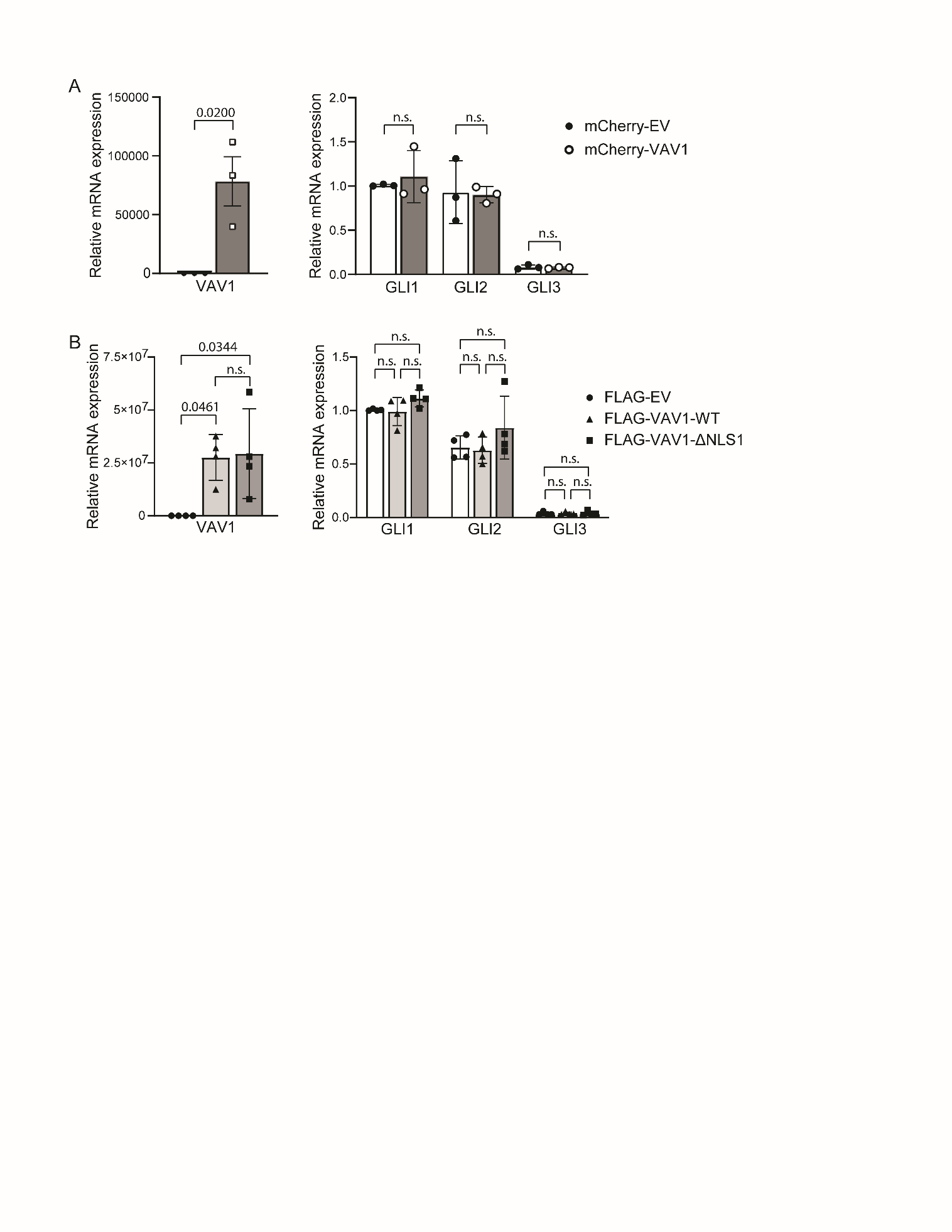


**Figure S2.** VAV1 overexpression does not alter mRNA expression of GLI family genes. (A) Relative mRNA expression of VAV1 (left) and GLI1, GLI2, and GLI3 (right) following overexpression of an mCherry-tagged wild-type VAV1 construct (n=3). Unpaired, 2-tailed t-tests performed for each gene comparing VAV1 overexpression to an empty vector control sample. (B) Relative mRNA expression of VAV1 (left) and GLI1, GLI2, and GLI3 (right) following overexpression of FLAG-tagged VAV1 constructs (WT, or ΔNLS1; n=4). One-way ANOVA was performed with a post hoc Tukey’s multiple comparison test. All data points indicate the average value for independent biological replicates.


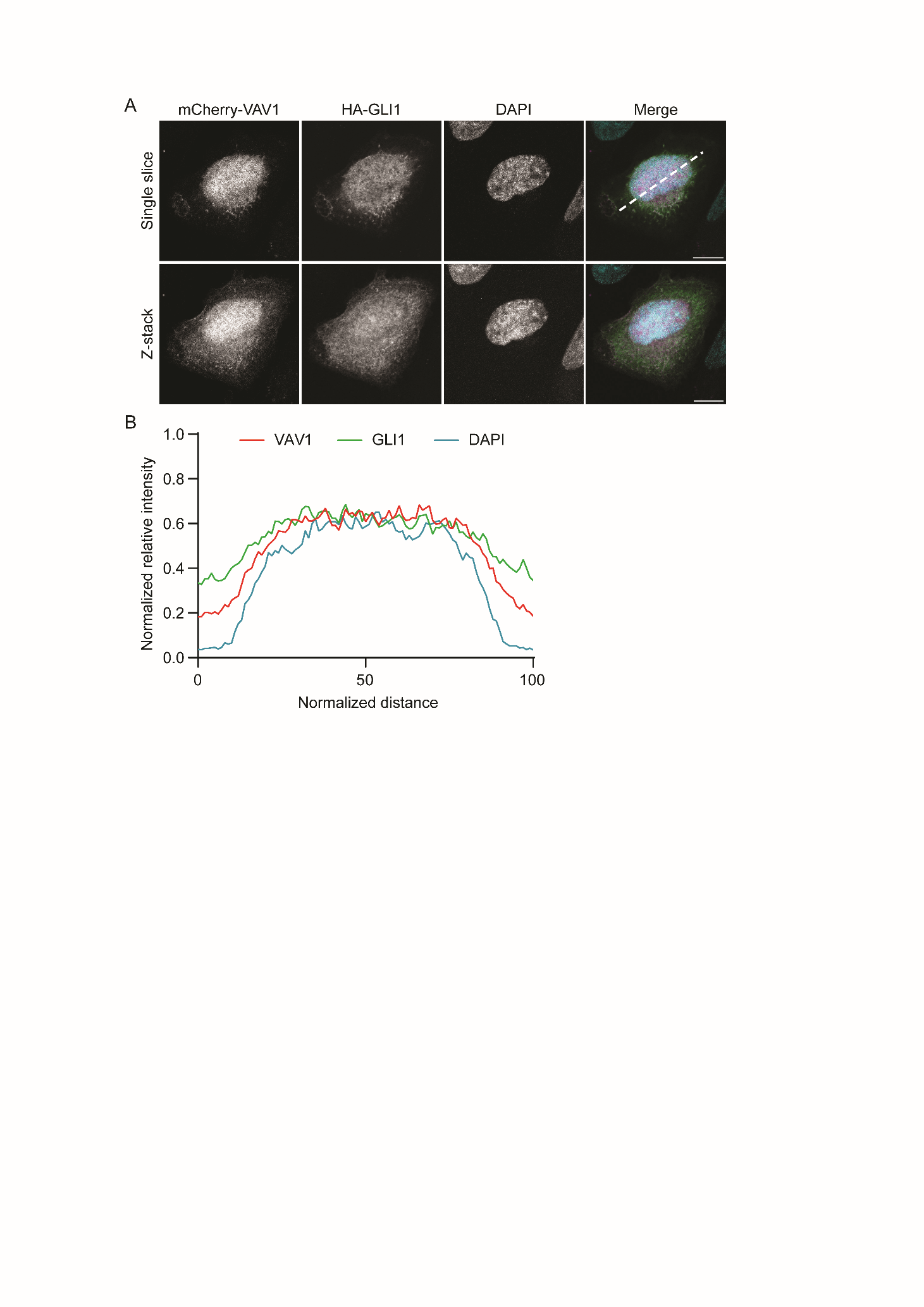


**Figure S3.** Overexpressed of VAV1-mCherry and HA-GLI1 colocalize in PDAC cells. (A) Representative single slice and Z-stack confocal microscopy image of mCherry-VAV1 (magenta) and HA-GLI1 (green) counterstained with DAPI (cyan) in PANC1 cells (n=3) (scale bar= 10 µm). The dashed line in A indicates where the line scan analysis was performed. (B) Line scan analysis profile plot showing colocalization of mCherry-VAV1 and HA-GLI1 compared to DAPI (n=30).


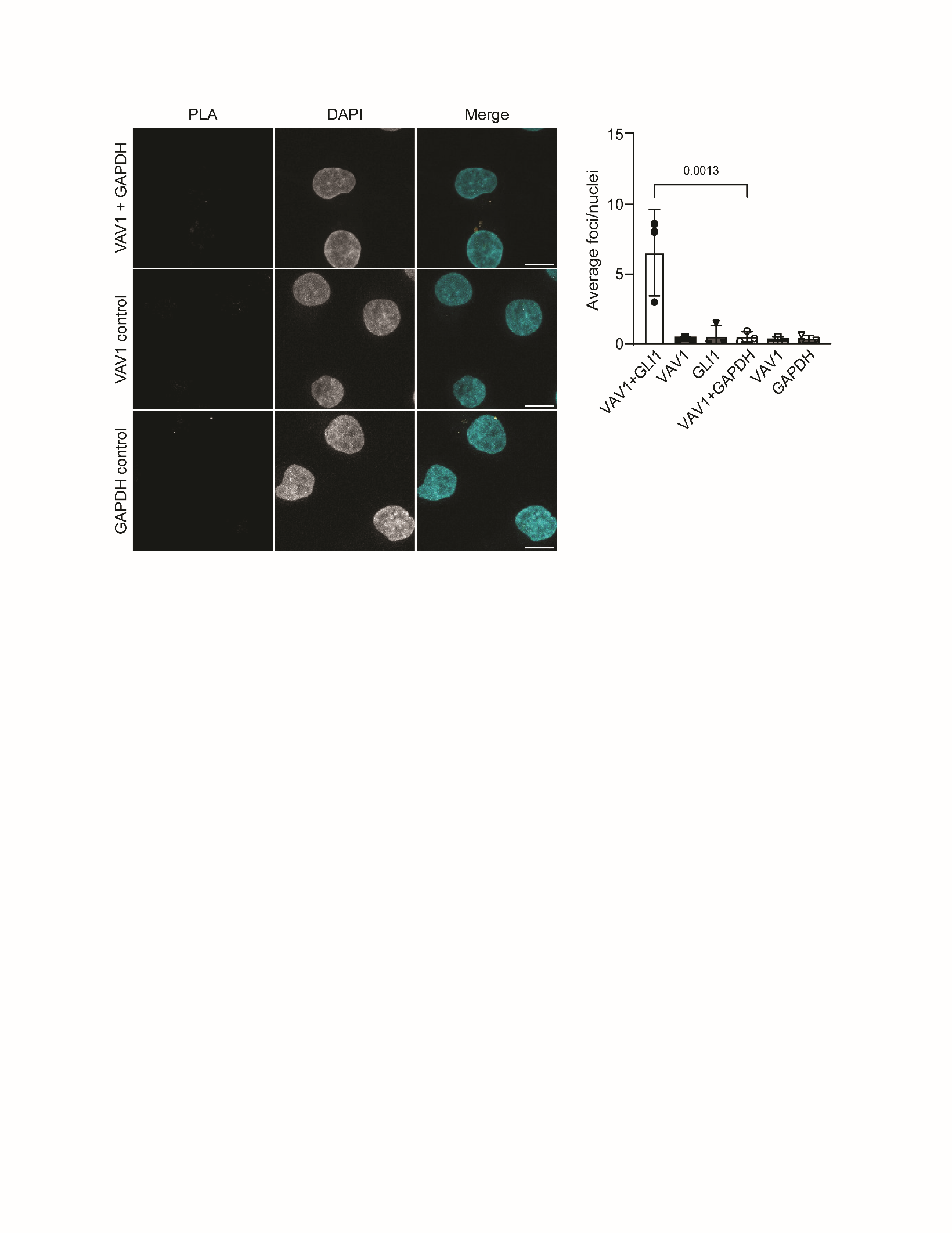


**Figure S4.** VAV1 PLA negative control using GAPDH. (A) Immunofluorescence images of PLA of VAV1 and GAPDH performed in Mia PaCa-2 cells. (scale bar= 10 µm). Left most three bars show PLA quantification data from Figure 4A. One-way ANOVA was performed with a Tukey’s test for multiple comparisons.


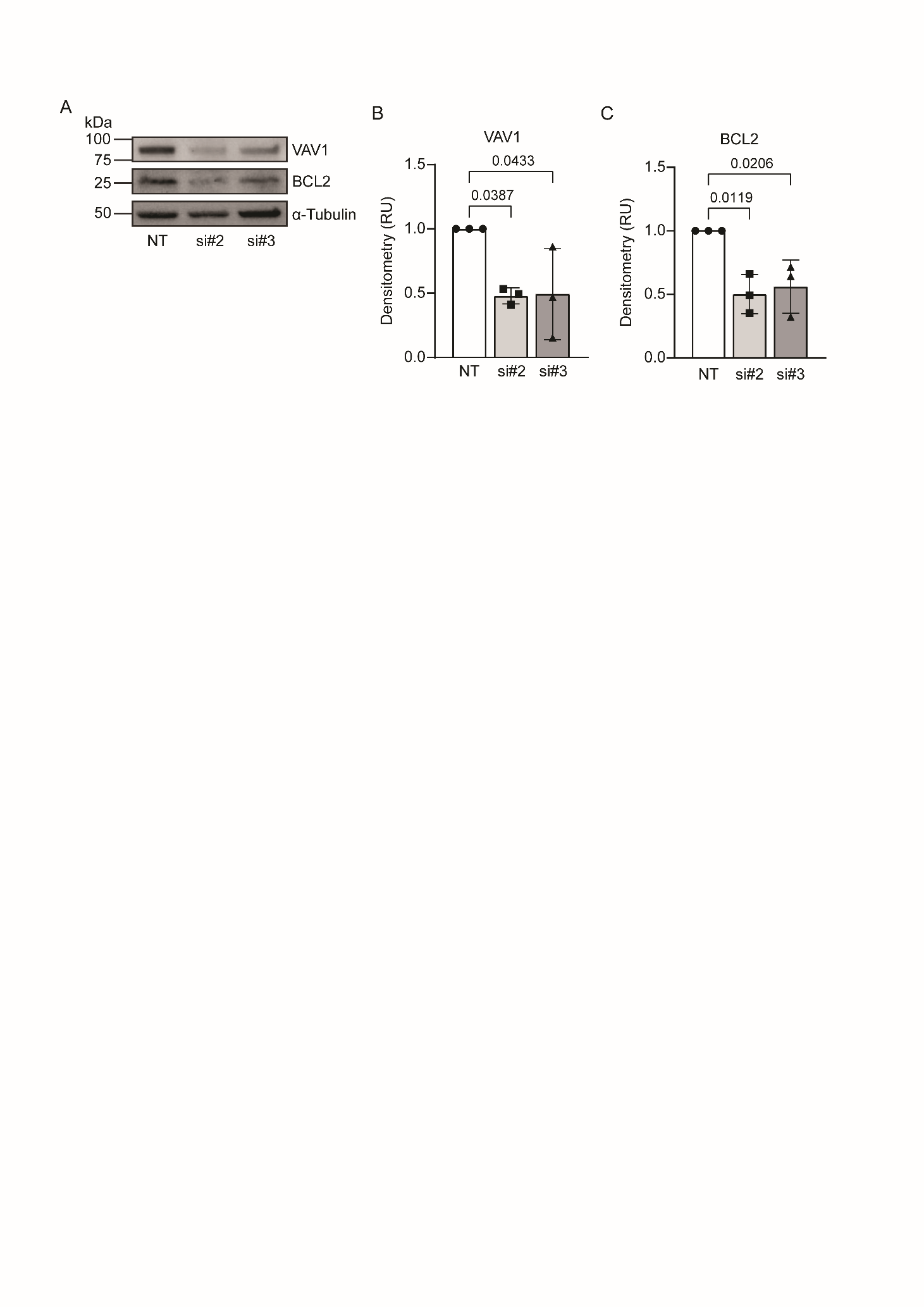


**Figure S5.** BCL2 expression in VAV1 knockdown cells using a second siRNA targeting sequence. (A) Representative Western blot images of VAV1 and BCL2 following siRNA knockdown of VAV1 using 2 independent VAV1 Qiagen siRNAs (n=3). (B-C) Densitometric analysis of VAV1 (B) and BCL2 (C) from Western blot images (n=3) using Fiji (ImageJ) software. One-way ANOVA was performed with a post hoc Dunnett’s multiple comparison test. All data points indicate an independent biological replicate.
